# Supplementary material for: A self-operating broadband spectrometer on a droplet
Source: Nat Commun. 2020 May 8;11:2263. doi: 10.1038/s41467-020-16206-8 (PMC7210994; doi:10.1038/s41467-020-16206-8)
Supplement: Supplementary file 1 — Supplementary Information [file 41467_2020_16206_MOESM1_ESM.docx]

*Supplementary informations for*:

A self-operating broadband spectrometer on a droplet

Malara et al.

The interferograms recorded by det1 and det2 (displayed in fig.1c of the main manuscript) consist of two arrays of *N* intensity values. The time spacing between successive interferogram datapoints is *dt*. A preliminary operation is to cut and reverse the arrays so that their first point corresponds to the instant when the liquid surface is totally evaporated. In order to extract the source spectrum by Fourier-transforming the recorded data, the operations described in the supplementary notes 1) and 2) are performed.

**Supplementary note 1**

**Transposing the interferogram in the spatial domain**. To obtain the FT spectrum in wavenumbers, the recorded time-domain interferograms must be expressed in a length scale. For this task, the basic notions to consider are that point #1 of both interferograms corresponds to *L=0* and one fringe of the reference interferogram corresponds to a physical displacement of the droplet surface $\Delta L=\frac{\lambda_{\mathrm{ref}}}{2n},$. In the following procedure, first, we assign a length axis to the reference interferogram (det2):

- define an array *ZDP* with all the positions of the zero derivative points of the reference interferogram. The half-fringe spacing between two successive points corresponds to half a wavelength of the reference radiation.
- For each point p of the reference interferogram find the i-th element of the *ZDP* array such that *ZDP(i)<p<ZDP(i+1)*.
- assign to point p the coordinate $L\left( p \right)=\left( i-1+\frac{ZDP\left[ i+1 \right]-p}{ZDP\left[ i+1 \right]-ZDP\left[ i \right]} \right)\frac{\lambda_{\mathrm{ref}}}{2n}$, that represents the instantaneous physical distance between the droplet surface and the fiber ferrule. The integer number (i-1) represents the number of half fringes between the first interferogram point and *p* (because the interferogram starts with a maximum, the first half fringe is completed at i=2); the second term describes the position of the point p as a fraction of the i-th half-fringe.

The length scale so obtained for the reference interferogram can be directly transferred to the signal interferogram. In fact, because the two signals are synchronous, the p-th point of the det2 interferogram has the same spatial coordinate of the p-th point of the det1 one. Once transposed in the length domain, the interferogram datapoints are not equally spaced (because they were recorded at a constant time separation *dt*, and evaporation is not constant in time). Last step to complete the x-scale data processing is therefore to interpolate the array, obtain a continuous function, then resample it with a fixed resolution. The det1 interferogram is then made of equally-spaced datapoints in the spatial domain.


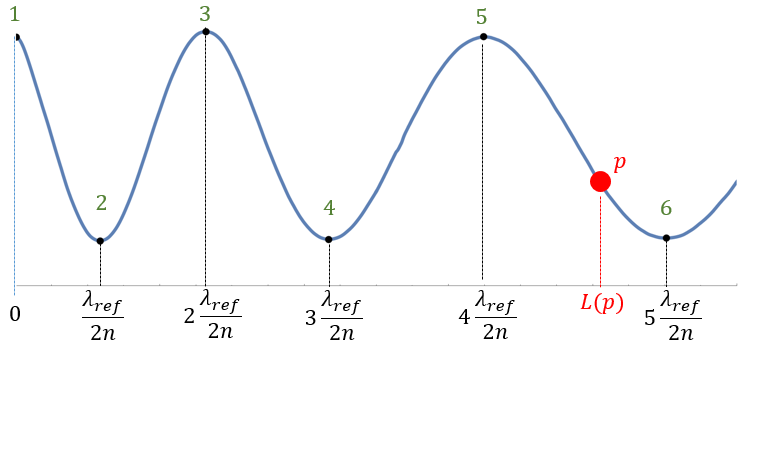


**Supplementary fig.1**: example of assignment of the coordinate L(p) to the p-th datapoint. The numbers in green represent the element# of the ZPD array. In this example, for the pth point, i=5, so $L\left( p \right)=\left( 4+\frac{ZDP\left[ 6 \right]-p}{ZDP\left[ 6 \right]-ZDP\left[ 5 \right]} \right)\frac{\lambda_{\mathrm{ref}}}{2n}$

It is worth remarking how the data recording and sampling of the described system is different from that of conventional FTIR spectrometers. In the latter instruments, the acquisition of the signal datapoints is triggered by the zero-crossing of the reference interferogram, so in each period of the reference radiation 2 signal datapoints are recorded. According to the Nyquist theorem, this allows to correctly sample only spectral components with a longer period in the interferogram. i.e. those with an wavelength longer than the reference (typically an 633 nm He-Ne laser).

Instead, in our system, the acquisition rate of datapoints is only set by the data acquisition board. This means that even the cheapest analog-to-digital converter allows plenty of oversampling. Indeed, consider that the reference interferogram traces around 700 fringes in two 2 minutes (5 fringes per second in average). According to the Nyquist criterium sampling that would require an acquisition bandwidth of only 10 Hz (10 points per second). By largely oversampling the reference interferogram and synchronously recording the signal, it is possible to correctly sample also spectral components faster (i.e. with a wavelength much shorter) than the reference radiation.

**Supplementary note 2**

**Making the interferogram independent of the droplet shape.** The signal interferogram, now in the length domain, can be written as :

$$I\left( L \right)=\int R_{1}I\left( k \right)dk+\int{C(L)R}_{2}T_{1}^{2}I\left( k \right)dk+\int\sqrt{C(L)T_{1}^{2}R_{1}R_{2}}\cdot I\left( k \right)\cos\left( 2kL \right)dk (1)$$

Supplementary eq.1 is the sum of three contributions that from now on will be referred to as A, B and C. The first goal is to isolate the fast and the slow components $I_{AC}\left( L \right)$ and $I_{DC}\left( L \right)$ from the recorded interferogram.

$I_{DC}\left( L \right)$ is generated by the first two terms of eq. (S1): $I_{DC}\left( L \right)=A+B$. During the evaporation, these terms contribute to the signal with a fixed and a slowly varying offset respectively. The term A corresponds to the total intensity reflected by the fiber-liquid interface alone, and can be easily assessed from the backreflection of the fiber when completely immersed in the liquid. For any practical purpose, A<<B when using isopropanol, so $I_{DC}\left( L \right)\cong B$.

The slowly varying offset B can be extracted from the signal interferogram by detecting its zero derivative points and building the arrays: *max(i)* and *min(i)*. The i-th array element is a 2-dimensional vector that maps position and signal level of the i-th maximum (minimum). The max and min arrays are then used to build the “MidPoints” array, where every point is defined as $MP\left( i \right)=\left\{ \frac{\min\left( i,1 \right)-\max\left( i,1 \right)}{2},\max\left( i,2 \right)-\frac{\max\left( i,2 \right)-\min\left( i,2 \right)}{2} \right\}$. Interpolation of the MidPoint array elements gives an experimental record of the term B(L), and it is shown as a red line in supplementary supplementary fig.2. By subtracting it from the interferogram, $I_{AC}\left( L \right)$ is obtained (inset of supplementary fig.2).


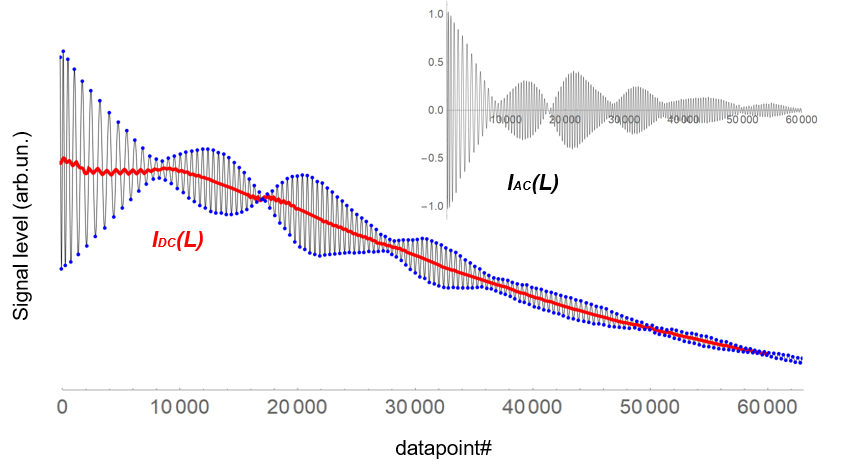


**Supplementary fig.2:** Extraction of the slow and fast signal components from the det1 interferogram: maxima and minima are detected (blue points) in order to build the Midpoint array (red curve), whose interpolation represents $I_{DC}\left( L \right)$ (in this example the procedure is applied before transposing to the length scale). Inset:$I_{AC}\left( L \right)$retrieved by subtracting the slow component.

$I_{AC}\left( L \right)$ corresponds to the term C of equation (S1), so it is equivalent to the Fourier transform of the source spectrum except for the factor $\sqrt{C(L)T_{1}^{2}R_{1}R_{2}}$. Now, while $T_{1}^{2}{, R}_{1}, R_{2}$depend only on the droplet constituent material, C(L) depends on the specific droplet shape and evaporation dynamics. In general, C increases as the droplet gets smaller, and produces a slowly increasing amplitude envelope in $I_{AC}\left( L \right)$ that is different from droplet to droplet.

It is possible to use the information contained in $I_{DC}\left( L \right)$ to remove the C(L) term dependence of $I_{AC}\left( L \right)$ before Fourier-transforming it. First step is to divide $I_{AC}\left( L \right)$ by the square root of $I_{DC}\left( L \right)$:

$$\frac{I_{AC}\left( L \right)}{\sqrt{I_{DC}\left( L \right)}}=\frac{\int\sqrt{C(L)T_{1}^{2}R_{1}R_{2}}\cdot I\left( k \right)\cos\left( 2kL \right)dk}{\sqrt{\int{C(L)R}_{2}T_{1}^{2}I\left( k \right)dk}} (2)$$

In the hypothesis that C(L) does not depend on the wavenumber k, the terms $\sqrt{C(L)}$ in the numerator and denominator can be extracted from the integrals and cancel out. The new signal becomes:

$$\frac{I_{AC}\left( L \right)}{\sqrt{I_{DC}\left( L \right)}}=\frac{\int\sqrt{T_{1}^{2}R_{1}R_{2}}\cdot I\left( k \right)\cos\left( 2kL \right)dk}{\sqrt{\int R_{2}T_{1}^{2}I\left( k \right)dk}} (3)$$

Now, the denominator represents the square root of the intensity reflected by the second surface into the fiber mode when C=1 (perfect coupling). This condition occurs at the very end of the evaporation process, when the droplet is just a thin layer, so all the intensity reflected by its outer boundary can be assumed to couple back into the fiber. The value $\int R_{2}T_{1}^{2}I\left( k \right)dk$ is thus just $I_{DC}\left( 0 \right)$. By multiplying for the square root of this factor we finally get:

$$I_{AC}\left( L \right) \sqrt{\frac{I_{DC}\left( 0 \right)}{I_{DC}\left( L \right)}}=\int\sqrt{T_{1}^{2}R_{1}R_{2}}\cdot I\left( k \right)\cos\left( 2kL \right)dk (4)$$

The recorded signal so manipulated is now equal to the Fourier transform of the source spectrum except for the factor $\sqrt{T_{1}^{2}R_{1}R_{2}}$, that depends only on the droplet material and not on its specific shape or evaporation dynamics. This factor can be accounted for as a fixed response function of the spectrometer.


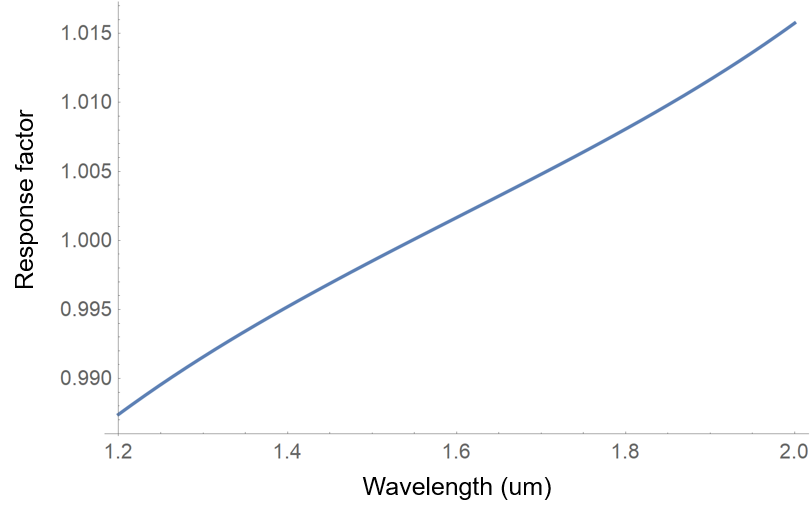


**Supplementary fig.3**: The very weak wavelength dependence of the factor $\sqrt{T_{1}^{2}R_{1}R_{2}}$ allows to approximate the Fourier transform of the AC2 signal to the spectrum of the radiation with an accuracy order of 1% across the whole NIR region.

The response $\sqrt{T_{1}^{2}R_{1}R_{2}}$ for isopropanol is plotted in supplementary fig.3 (rescaled to 1) as a function of wavelength. The refractive indexes *n_iso_ * and n_air_** of isopropanol and air were evaluated using Sellmeier equations [1,2] while the fiber effective refractive index *n_fiber_ *was calculated starting from the group-index values reported in the specs sheet of the Corning SMF28 optical fiber. These dispersion curves allow to calculate the Fresnel reflectivities/transmissivities $R_{1}(\lambda)$, $R_{2}(\lambda)$ and $T_{1}^{2}(\lambda)$ and therefore the wavelenght dependence of the spectrometer response function. The plot of supplementary fig.3 clearly shows that, besides a scaling factor, the $\sqrt{T_{1}^{2}R_{1}R_{2}}$ is flat to the 1% tolerance in all the NIR window. The FT of the AC_2_ (L) interferogram is therefore an accurate approximation of the actual spectrum of the radiation, as can be easily seen in fig.2b of the main manuscript.

**Supplementary References**

[1] P. E. Ciddor*. “Refractive index of air: new equations for the visible and near infrared”,* [Appl. Optics **35**, 1566-1573 (1996)](https://doi.org/10.1364/AO.35.001566)

[2] E. Sani, A. Dell’Oro, “*Spectral optical constants of ethanol and isopropanol from ultraviolet to far infrared”*. Opt. Mater. **60**, 137–141 (2016)
